# Fully Integrated High-Performance MEMS Energy Harvester for Mechanical and Contactless Magnetic Excitation in Resonance and at Low Frequencies

**DOI:** 10.3390/mi13060863

**Published:** 2022-05-30

**Authors:** Mani Teja Bodduluri, Torben Dankwort, Thomas Lisec, Sven Grünzig, Anmol Khare, Minhaz Ahmed, Björn Gojdka

**Affiliations:** Fraunhofer Institute for Silicon Technology ISIT, Fraunhoferstrasse 1, 25524 Itzehoe, Germany; mani.teja.bodduluri@isit.fraunhofer.de (M.T.B.); thomas.lisec@isit.fraunhofer.de (T.L.); sven.gruenzig@isit.fraunhofer.de (S.G.); anmol.khare@isit.fraunhofer.de (A.K.); minhaz.ahmed@isit.fraunhofer.de (M.A.); bjoern.gojdka@isit.fraunhofer.de (B.G.)

**Keywords:** piezoelectric vibrational energy harvester, MEMS, frequency up-conversion, wafer-level integrated magnets, contactless magnetic coupling, rotational harvesting, high-density proof mass, AlN

## Abstract

Energy harvesting and storage is highly demanded to enhance the lifetime of autonomous systems, such as IoT sensor nodes, avoiding costly and time-consuming battery replacement. However, cost efficient and small-scale energy harvesting systems with reasonable power output are still subjects of current development. In this work, we present a mechanically and magnetically excitable MEMS vibrational piezoelectric energy harvester featuring wafer-level integrated rare-earth micromagnets. The latter enable harvesting of energy efficiently both in resonance and from low-g, low-frequency mechanical energy sources. Under rotational magnetic excitation at frequencies below 50 Hz, RMS power output up to 74.11 µW is demonstrated in frequency up-conversion. Magnetic excitation in resonance results in open-circuit voltages > 9 V and RMS power output up to 139.39 µW. For purely mechanical excitation, the powder-based integration process allows the realization of high-density and thus compact proof masses in the cantilever design. Accordingly, the device achieves 24.75 µW power output under mechanical excitation of 0.75 g at resonance. The ability to load a capacitance of 2.8 µF at 2.5 V within 30 s is demonstrated, facilitating a custom design low-power ASIC.

## 1. Introduction

Energy harvesting systems are promising solutions for powering smart autonomous electronic systems such as devices for the internet of things (IoT), wearable electronics, and wireless sensor nodes. Moreover, advances in miniaturization have considerably reduced the power consumption of such microsystems. Today, such devices require power in the range of micro to milliwatts. Light (photovoltaic cells) [1] and thermal gradients (thermo-generators) [2,3] are some of the sources of ambient energy which are already commercially utilized to power devices. Contrarily, harvesters scavenging energy from mechanical vibration-based sources have not had the same commercial success up to now. However, the required amount of power can be provided by micro-scale energy harvesting based on microelectromechanical systems (MEMS) technology. In conjunction with corresponding power management electronics, such MEMS can provide an integrated, miniaturized power supply for autonomous sensor networks [4,5].

**Figure 1:**
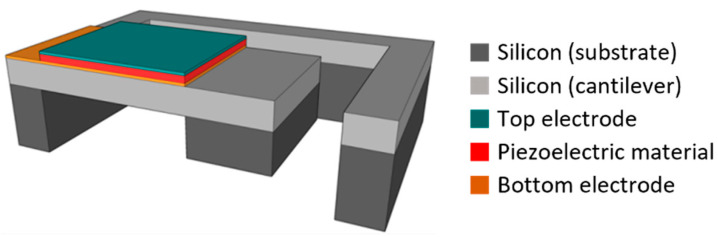
Schematic illustration of a MEMS piezoelectric vibrational energy harvester. Typical designs comprise a movable structure (cantilever) made of silicon with a piezoelectric transducer patch on top and a passive tip mass made of silicon.

MEMS technology is well suitable to fabricate cost-efficient miniaturized vibrational energy harvesters in large quantities. Electrostatic [6,7], electromagnetic [8,9,10] and piezoelectric transduction mechanisms [11,12,13] are the most employed in such energy harvester systems. Among them, the piezoelectric-based harvesters represent promising candidates due to their high power densities [14,15]. Figure 1 presents a schematic view of a typical MEMS-based piezoelectric vibrational energy harvester. Such a single clamped construction yields comparatively low resonance frequencies and a high bending moment, which induces high stress in the piezoelectrical material. It basically comprises a movable structure (cantilever) with a passive tip mass made from silicon and a transducer, made of a piezoelectric layer with top and bottom electrodes. Such designs suffer from two drawbacks. Firstly, significant power is generated only within a narrow frequency band around the harvester’s resonance frequency [16]. Accordingly, it is necessary to match the resonance frequency of the device to the excitation source. The device must be optimized for specific applications or more complex designs are necessary to adapt the resonance frequency [17,18,19]. However, in MEMS technology, only specific devices with predetermined frequencies can be fabricated at low costs based on batch fabrication. Secondly, the resonance frequencies remain well above the frequencies of most ambient vibrations (<200 Hz) due to a low effective tip mass resulting from the low mass density of silicon [4].

To overcome those problems, frequency up-conversion techniques have been proposed [18]. In the case of up-conversion, the movable cantilever vibrates at its resonance frequency after a low frequency external excitation. To induce sufficient initial deflection, high impulse-like forces are necessary. Physical contact-based excitation on the tip mass can be utilized for frequency up-conversion. In this case, the harvester is excited by external physical pulses from, e.g., a second cantilever [20]. However, stability issues arise due to the direct physical contact, which limits applicability in the long run.

With a permanent magnet as tip mass instead of the silicon mass, non-contact excitation as well as an increased tip density are achieved simultaneously [21,22]. The magnetic coupling enables energy harvesting from rotational and translational motions, which extends the range of applications significantly. It has been demonstrated that frequency up-conversion strategies based on magnetic plucking can enhance the harvester performance at low excitation frequencies [23,24,25]. Furthermore, magnets allow tuning of the harvester resonance frequency [26] or the creation of bistable systems with increased power output [27,28]. Furthermore, in the case of common mechanical excitation, an increased tip mass from compact high-density materials enables higher energy output at the same level of vibrations. However, a MEMS device with wafer-level integrated rare-earth magnets or high-density tip materials has not been available up to now. Known implementations are assembled as hybrids, for instance commercial magnets are manually glued to the cantilever [29]. That drives up the device costs significantly and is incompatible with MEMS fabrication techniques. Recently, a novel method for the wafer-level integration of micron-sized structures of various shapes and sizes and of different materials has been developed. The so-called PowderMEMS technique is compatible with standard MEMS processes [30,31]. Using PowderMEMS, a piezoelectric vibrational energy harvester with wafer-level integrated, rare earth permanent micromagnets was demonstrated [32,33,34]. To the best of our knowledge, this is the first MEMS energy harvester featuring integrated rare-earth magnets, which is completely fabricated on the wafer-level by MEMS and semiconductor processes, allowing large-scale production.

In this paper, both magnetic and mechanical excitation of such devices is investigated and compared. In Section 2, design considerations are explained. A detailed description of the fabrication process is provided in Section 3. The unique wafer-level integration of micromagnets for MEMS systems is highlighted. The experimental setups used for the investigation are introduced in Section 4. The output characteristics of the harvester are investigated and discussed in Section 5. Both mechanical and magnetic excitation is applied in resonance and for low frequencies in frequency up-conversion. Furthermore, a demonstrator with a custom-designed application-specific integrated circuit (ASIC) for rectification and capacitors for energy storage are presented.

## 2. Design Considerations

**Figure 2:**
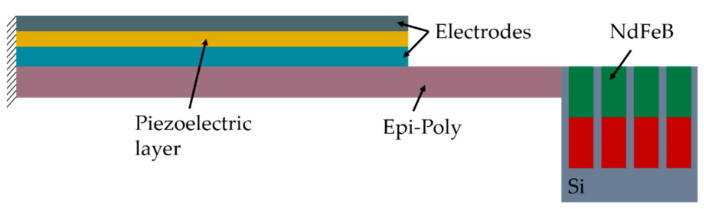
Simplified cross-section of the MEMS piezoelectric vibrational energy harvester with integrated NdFeB micromagnets (refer to Section 3 for more details).

The proposed MEMS harvester generally follows the widely used design of a movable cantilever with a piezoelectric transducer [35]. Figure 2 schematically illustrates the cross-section of the device (for a detailed cross-section, see Section 3). Polycrystalline silicon (Epi-Poly), which can be deposited in an epitaxial reactor with thicknesses between 2 µm and 80 µm is used as the passive layer material of the cantilever. It exhibits mechanical properties very similar to monocrystalline silicon. The piezoelectric transducer on top of the Epi-Poly consists of AlN as the piezoelectric material and two metal electrodes. The key difference between conventional devices, as shown in Figure 1, and the harvesters reported here are the integrated NdFeB micromagnets, occupying the bulk of the silicon tip mass.

The major factors that influence the output power P are the piezoelectric material, the geometry of the harvester, the excitation force F and the damping characteristics of the system (electrical $\zeta_{e}$ and mechanical $\zeta_{m}$ damping ratios). Correspondingly, when the resonance frequency of the harvester matches the excitation frequency the maximum generated power is given by [13]

(1) $$P=\frac{\zeta_{e}F^{2}}{4f_{0}m{\left(\zeta_{e}+\zeta_{m}\right)}^{2}}$$

To investigate the influence of the excitation force, three designs with varying tip magnet dimensions are fabricated. The tip mass is increased within a constant volume compared to silicon due to the higher density of NdFeB. The larger effective mass m of the movable structure results in a reduced resonance frequency $f_{0}$, following [36]

(2) $$f_{0}=\frac{1}{2\pi }\sqrt{\frac{k}{m},}$$

where k is the stiffness of the cantilever. The NdFeB micromagnets exhibit high magnetic flux densities allowing for magnetic coupling with external fields. The magnetic excitation force $F=\mu_{0}\mathrm{VM}\nabla H$ can be scaled by increasing the volume V of the tip magnet, with M being the magnetization and H the external magnetic field strength. Additionally, in the case of mechanical excitation, the increased mass leads to increased inertia. Consequently, at a certain acceleration level, higher output voltages can be achieved compared to a conventional silicon tip mass [37].

**Figure 3:**
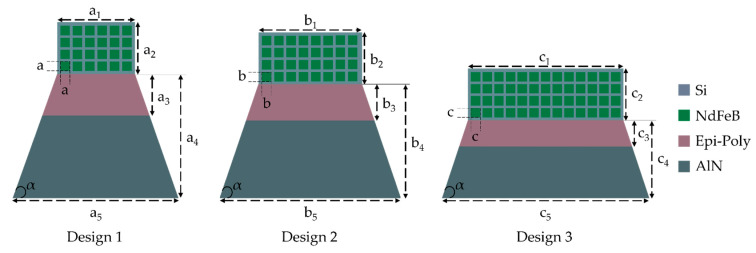
Schematic top view of the piezoelectric energy harvester designs with integrated NdFeB micromagnet arrays (corresponding cross-sections given in Figure 2). The angle α=70° is kept constant in all designs.

Figure 3 illustrates the harvester designs investigated in this work. It was demonstrated that a trapezoidal cantilever yields better power output and device reliability compared to traditional rectangular designs [34]. Thus, an isosceles trapezoidal cantilever structure is used in all three designs with a base angle α=70° at the clamped end. The thicknesses of the material layers remain unvaried across the designs: 2 µm piezoelectric AlN, 29 µm poly-Si, 500 µm NdFeB-micromagnets, 100 nm Mo top and 130 nm Ti/Pt bottom electrodes.

Stress distribution profiles were simulated for the different designs using the structural mechanics module in COMSOL Multiphysics (Version 6, Göttingen, Germany). The chosen material properties from the COMSOL material library are summarized in Table 1 and Table 2. The thin film top and bottom electrodes are neglected in the mechanical simulation as their thickness is less than 1% of the whole cantilever stack thickness.

**Figure 4:**
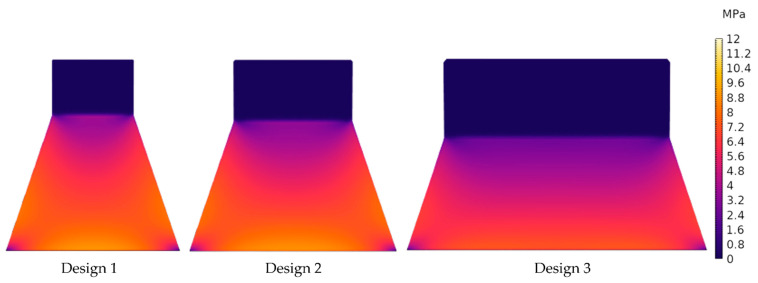
FEM simulated von Mises stress contour plots for the three harvester designs.

A stationary analysis was performed with the clamped end of the cantilever being fixed as a constraint boundary condition. The excitation is provided by application of a boundary force to the tip mass with values of 1.3 mN, 1.6 mN and 2.2 mN for the designs 1, 2 and 3, respectively (the force calculations are discussed in more detail in Section 5.1). The resultant stress profiles are shown in the Figure 4. The stress profiles exhibit a non-uniform stress distribution across the cantilever. Accordingly, if the top and bottom electrodes cover the whole area of the cantilever, this non-uniformity results in charge redistribution. Consequently, the average voltage is reduced due to equalization as the charges flow from highly stressed regions to areas with lower stress [38].

To circumvent this problem, the harvester design is optimized by confining the area of the piezo stack to the uniformly distributed stress area, as shown in Figure 3. The volume of the integrated micromagnet array and the main geometric parameters are summarized in Table 3. Please note that the volumes of the movable part were calculated considering the Epi-Poly and the tip mass only. Due to the comparably low thickness of the piezo patch, its contribution to the volume was neglected. Furthermore, the silicon window area and die-area for all samples are 16 mm^2^ and 48 mm^2^.

## 3. Fabrication Process

**Figure 5:**
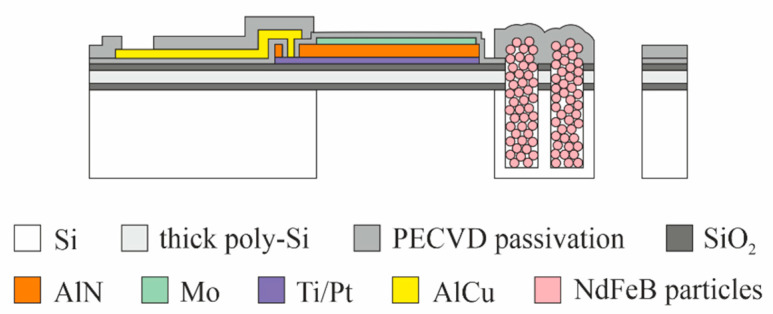
Schematic cross-section of the MEMS piezoelectric energy harvester with integrated NdFeB-based micromagnets.

The piezoelectric energy harvesters are fabricated on 8-inch silicon substrates. In total, nine lithography layers (photomasks) are needed. Figure 5 presents a schematic cross-section through the finished device. A brief description of the process flow is given below.

After the substrate is oxidized (650 nm SiO_2_), a poly-crystalline silicon film of 29 µm thickness, acting as passive layer material, is deposited in an epitaxy reactor. The poly-Si is then covered with 1 µm of SiO_2_ using low pressure chemical vapor deposition (LPCVD). On top of the oxide, the piezo stack is deposited: 30 nm Ti/100 nm Pt (bottom electrode) are evaporated, followed by 2 µm of sputtered AlN (piezoelectric material) and 100 nm of sputtered Mo (top electrode). With the first three lithography layers, the piezo stack is patterned top-down by reactive ion etching (RIE) and wet etching processes. After the deposition of 1 µm silicon nitride by plasma-enhanced chemical vapor deposition (PECVD), the 4th lithography contact holes to the bottom and top electrodes of the piezo stack are defined in the passivation layer by RIE. Sputtering of 1 µm AlCu_0.5_ alloy is followed by the 5th lithography and the patterning of the metal by wet etching.

**Figure 6:**
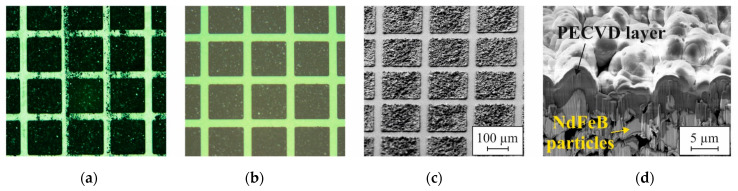
Photographs of a micromagnet array (**a**) after agglomeration of the NdFeB particles and (**b**) after surface conditioning. The SEM micrograph in (**c**) shows a micromagnet array after passivation with 3 µm PECVD silicon oxide and (**d**) a FIB cross-section through such a pixel.

For the subsequent integration of the micromagnets, a thick layer of photoresist is applied and, after the 6th lithography, cavities of about 500 µm depth are etched into the substrate by deep reactive ion etching (DRIE). Without removing the photoresist mask, the wafers are now transferred out of the cleanroom into a dedicated PowderMEMS lab. NdFeB hard magnetic powder (Magnequench MQFP-B+-10215-089, D50 = 5 µm) is dry filled into the cavities using a custom-developed automatic mold filling process [39]. Then, the loose dry powder is agglomerated into rigid 3D microstructures by 75 nm Al_2_O_3_ deposited by ALD at 75 °C. Figure 6a depicts a photograph of such a micromagnet array after agglomeration. As can be seen, many NdFeB particles remain on the substrate surface outside the cavities. Therefore, to enable post-processing in the cleanroom, a surface conditioning procedure is applied [30,40]. Grinding and polishing is used to remove particles from the backside of the substrate. For frontside cleaning, the photoresist is lifted off in O_2_ plasma and organic solvents. Figure 6b presents a photograph of a micromagnet array after surface conditioning.

After transfer back into the cleanroom, the micromagnets are passivated with 3 µm silicon oxide by PECVD to prevent intrusion of liquids into the porous structures during the subsequent processing. An SEM micrograph of a passivated micromagnet array is shown in Figure 6c. A FIB cross-section through such a pixel is displayed in Figure 6d. After the 7th lithography, the PECVD passivation is patterned by RIE to expose the bond pads. Then, after the 8th lithography, the passive layer stack, consisting of the thick polysilicon embedded between SiO_2_ layers, is patterned by RIE and DRIE. The last step of the fabrication process is the release of the movable structure by DRIE from the backside of the substrate after the 9th lithography.

**Figure 7:**
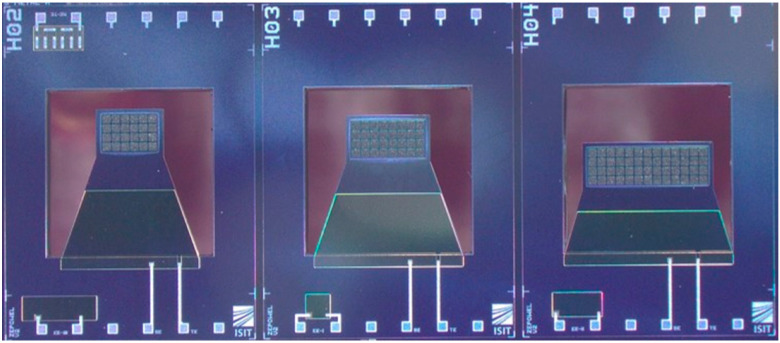
Photograph of different energy harvester dies before dicing. The three different designs were used for comparison in this study.

Finally, the micromagnets are magnetized on the wafer-level. A custom-made magnetization tool is utilized (MAGSYS, Dortmund, Germany) which provides a homogeneous magnetic field of 3.5 T perpendicular to the substrate surface over the area of a 200 mm wafer. An optical image of the final devices is depicted in Figure 7.

## 4. Experimental

### 4.1. Measurement Setups for Magnetic Excitation

**Figure 8:**
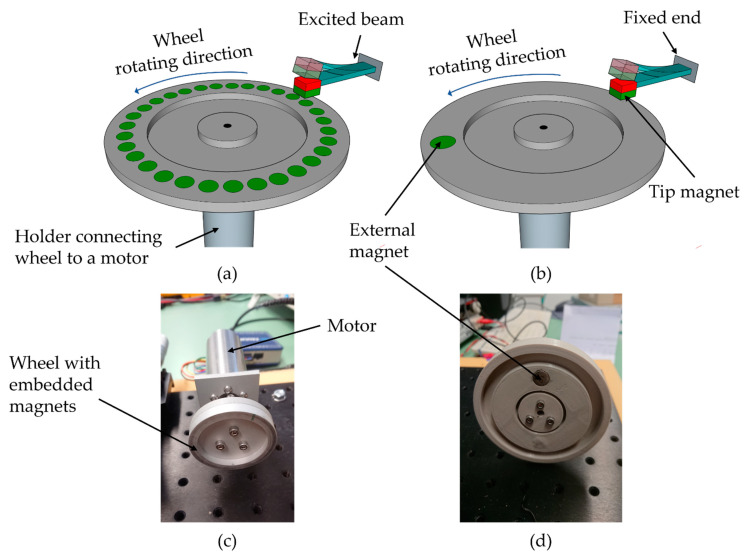
Schematic illustration of the setups utilized for magnetic excitation (**a**) in resonance and (**b**) in frequency-up conversion. Optical images showing the excitation wheels: (**c**) with 32 embedded magnets (underneath an aluminum cover) as used for resonant excitation; (**d**) the pole wheel with single magnet as used for magnetic plucking is shown without cover.

Magnetic excitation of the harvesters was applied in two different rotating wheel setups, as shown in Figure 8. The configuration in Figure 8a enables excitation in resonance whereas low-frequency magnetic plucking with frequency up-conversion was performed with the setup from Figure 8b. The tip mass of the harvester is colored in red and green to denote the vertical magnetization of the integrated micromagnets. The green color of the excitation magnets, embedded within the rotating wheel, illustrates that excitation occurs in repulsive force mode to maximize the energy output [22].

For resonance excitation, one wheel (Figure 8c) was equipped with 32 N52 NdFeB rod magnets with a diameter of 1.7 mm and a length of 6.6 mm. Each of the magnets was fitted into a blind hole within the wheel. A cover made of aluminum with a thickness of 1 mm was attached to the wheel to prevent release of the magnets during wheel rotation. The distance d refers throughout this article to the distance of the magnet used for exciting the harvester and the cantilever itself.

The second wheel was equipped with a single magnet as depicted in Figure 8d. A NdFeB N52 rod magnet with a diameter of 10 mm and a length of 10 mm was used in this case.

**Figure 9:**
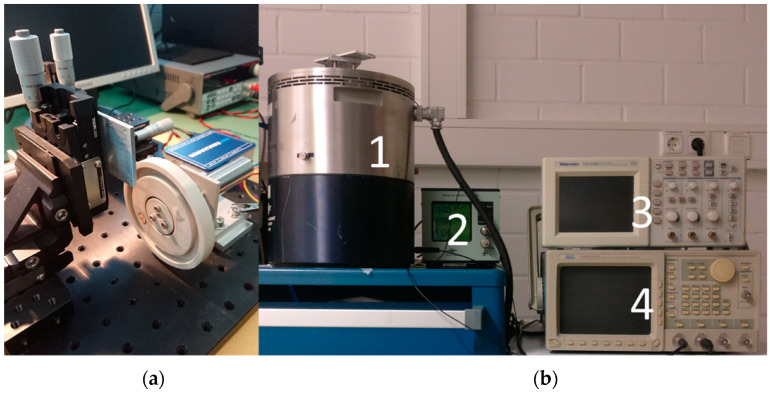
(**a**) Photograph of the measurement setup for magnetic excitation with pole wheel, motor and stage. The photograph in (**b**) presents an overview of the shaker setup with high displacement head (1), charge amplifier (2), oscilloscope (3) and frequency generator (4).

Both pole wheels were driven by a DC motor (2264W012BP4 and 3274G024BP4, Faulhaber, Schönaich, Germany) with the motor speed being precisely controlled by a motion controller (MC 5010s, Faulhaber). The harvester chip was assembled to PCB and clamped to a 3-axis translation stage (XRN25P-K2/M, Thorlabs, Newton, NJ, USA) to enable precise positioning of the harvester opposite the pole wheel, Figure 9a.

### 4.2. Measurement Setup for Mechanical Excitation

For mechanical excitation of the harvester, the shaker setup depicted in Figure 9b was utilized. As in the case of magnetic excitation, two distinct excitation modes were evaluated. In the first mode, sinusoidal signals with varied frequency were used to actuate the harvester in resonance. Since the acceleration of most ambient sources of vibration is well below 1 g [16,41], the applied excitation was restricted to this limit. In the second mode, pulse signals with 10% duty cycle were applied to mechanically trigger frequency up-conversion. In this case, the acceleration was set to 51 g, which corresponds to values typical for applications such as tire pressure monitoring [42].

The core components of this setup are as follows: Exciter body (Brüel & Kjær Type 4805, Nærum, Denmark), high displacement head power amplifier (Brüel & Kjær Type 2707), charge amplifier (Brüel & Kjær Type 2525), reference accelerometer sensor (Brüel & Kjær DeltaTron sensor Type 4396), NI data acquisition device (DAQ 6120, BNC-2110, Austin, TX, USA), digital oscilloscope (Tektronix MDO3034, Beaverton, OR, USA) and waveform generator. The acceleration was monitored using the reference accelerometer in conjunction with the charge amplifier. The PCB with the harvester chip was always mounted on a holder, which was attached to the membrane of the high displacement head.

The quality factors Q of the harvesters were measured from frequency sweep curves V(f) with

(3) $$Q=\frac{f_{0}}{\mathrm{BW}}\;$$

where the bandwidth $\mathrm{BW}=f_{2}-f_{1}$ is calculated based on −3 dB gain points, with $f_{1}$ and $f_{2}$ being the lower and upper frequencies, for which $V\left(f_{1,2}\right)=\frac{V_{\max}}{\sqrt{2}}\;$ holds and $V_{\max}$ (0 dB) is the voltage at the resonance frequency.

To determine the power output under the resonant condition, the harvesters were excited at their respective resonance frequency. The optimal load $R_{L}$ was measured by varying the load resistance. It should be noted that the load resistance is actually complex. In the case of resonance, only the ohmic resistance can be considered as by definition the phase shift is zero [43]. The RMS voltage $V_{\mathrm{RMS}}$ was measured and the RMS power output $P_{\mathrm{RMS}}$ was calculated according to Ohm’s law

(4) $$P_{\mathrm{RMS}}={\left(V_{\mathrm{RMS}}\right)}^{2}/R_{L}.$$

### 4.3. Rectification and Energy Storage

For rectification of the generated current, a low-power ASIC was used (designed by Fraunhofer IPMS, Dresden, Germany and produced by X-FAB Silicon Foundries, Erfurt, Germany). The ASIC is designed to be powered by the energy harvester itself, but it can optionally also be externally powered. The ASIC was specifically designed to accept 1–5 $V_{\mathrm{ac}}$ input signals, leading to output of 0–5 $V_{\mathrm{dc}}$ voltage and 0–50 µW power. A detailed description of the ASIC design and a detailed performance analysis is beyond the scope of this work and will be covered in a future study.

**Figure 10:**
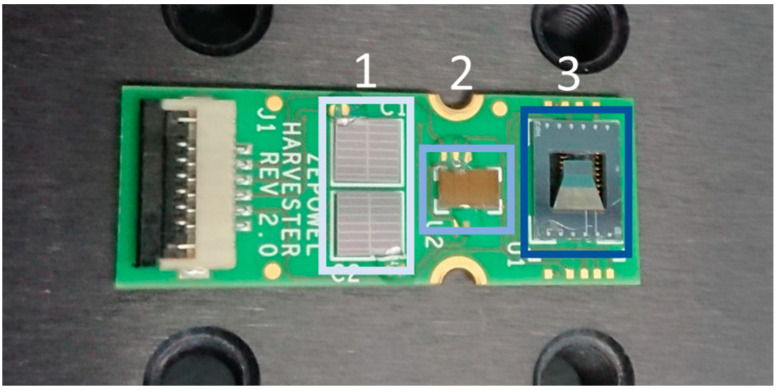
Photograph of a complete module comprising of two capacitors (1.4 µF each) (1), one ASIC (2) and one design 2 harvester (3).

For storage of the harvested energy, metal-insulator-metal capacitors were used, which were designed and realized by Fraunhofer IPMS (Dresden, Germany). Single capacitors of 4 × 4 mm were fabricated with a capacitance of 1.4 µF. Two such capacitors together with a harvester and an ASIC were mounted on a PCB, as shown in Figure 10, to form a micropower supply including energy harvesting, rectification, and energy storage.

## 5. Results

The fabricated harvesters were characterized using the setups described in Section 4. Section 5.1 and Section 5.2 focus on the performance of the harvesters considering magnetic and mechanical excitation under resonant conditions. The experimental data are compared to predictions from FEM simulations to validate the results. In Section 5.3, the performance of the harvesters is evaluated for low-frequency magnetic and mechanical excitation in frequency up-conversion. Finally, Section 5.4 demonstrates the harvesting, rectification and storage of the output achieved under excitation in frequency up-conversion.

### 5.1. Magnetic Excitation in Resonance

**Figure 11:**
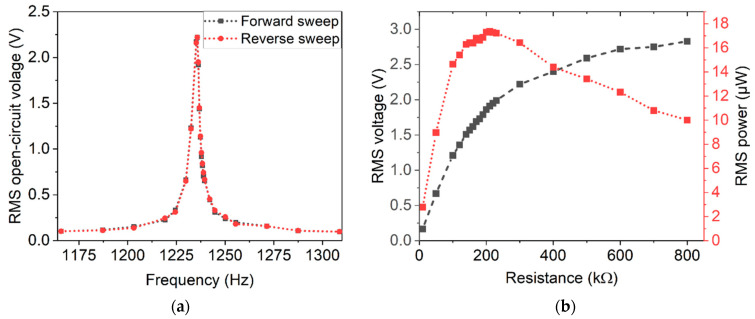
Representative measurements of open-circuit voltage in dependence of the excitation frequency, close to the resonance frequency of a design 2 harvester. (**a**) The resonance curve for a forward and a backward frequency sweep. (**b**) Typical measurements of the voltage and power output of a harvester with respect to applied ohmic loads.

To determine the resonance frequencies, the open-circuit voltage $V_{\mathrm{oc}}$ was measured in dependence of the excitation frequency. A typical measurement, including a forward and a reverse frequency sweep, is depicted in Figure 11a. Three devices of each design were characterized. The sweep measurements were performed at 4 mm distance between harvester and excitation magnets. At that distance, the devices exhibit symmetric resonance peaks and no resonance shift between the forward and backward sweep was observed. Hence, the excitation occurred in the linear response regime [44]. The measured resonance frequencies are in good agreement with the values predicted by FEM simulations, as summarized in Table 4.

A typical measurement of the voltage output and the corresponding calculated power output as a function of the load resistance is depicted in Figure 11b. Since the internal resistance of the harvesters typically reach more than 150 kΩ, the internal resistance of the used oscilloscope needs to be considered. Thus, both internal resistances were considered in a parallel connection for the calculation of the output power. The maximum in the power curve indicates optimal load resistance. In Table 4, a summary of mean values and standard deviations for the determined internal resistance is given for each design.

**Figure 12:**
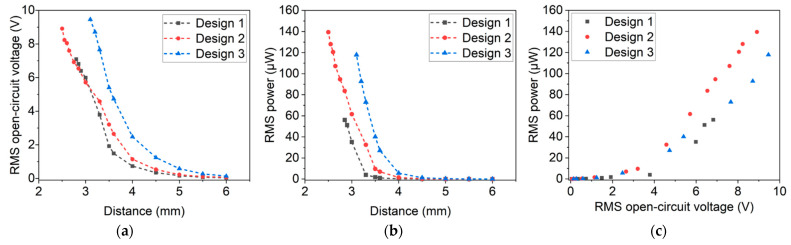
(**a**) Open-circuit voltage and (**b**) power output with respect to distance between harvester and excitation magnets. (**c**) Overview of achieved RMS open-circuit voltage and RMS power output at optimal load for the different harvester designs. (The different designs are introduced in Section 3).

The harvester’s output characteristics were further studied by measuring the open-circuit voltage at varying distances (see Figure 12a). Independent of the design, open-circuit voltages up to more than 6 V could be obtained. From a performance point of view, design 1 and design 2 achieve similar outputs. Design 3, featuring the largest micromagnet array, generates the highest voltages. However, it is especially sensitive to fracture and can be operated only at larger distances. Depending on the specific design, the probability of failure increased due to higher magnetic excitation forces at distances below 3 mm. Possible reasons for those failures are excessively high oscillation amplitudes or twisting of the cantilever due to the transversal approach of the excitation magnets. A detailed analysis regarding failure modes and (long term) stability is beyond the scope of this article and will be the subject of future work. In summary, design 2 harvesters exhibit the highest resistance to fracture, combined with high voltage and power output.

In a similar procedure, the power output at optimal resistance was determined (see Figure 12b). Design 2 harvesters exhibited the highest performance, achieving values of up to 139.39 µW. Harvesters of design 3 and design 1 exceeded power outputs of 117.85 µW and 56.08 µW, respectively.

In Figure 12c, the measurement results are summarized by plotting the open-circuit voltage against the power output. As expected from Equation (4), a quadratic dependency between open-circuit voltage and power output is observed. These results highlight that in resonance RMS, open-circuit voltages and RMS power outputs of >9 V and >130 µW, respectively, can be achieved.

FEM simulations of magnetic excitation were performed to validate the observed measurements. To reduce the meshing complexity and computational time, the modeling is divided in two steps. At first, the magnetic force between the tip magnet and external magnet is simulated and secondly, the calculated force values are used as input for the structural model.

**Figure 13:**
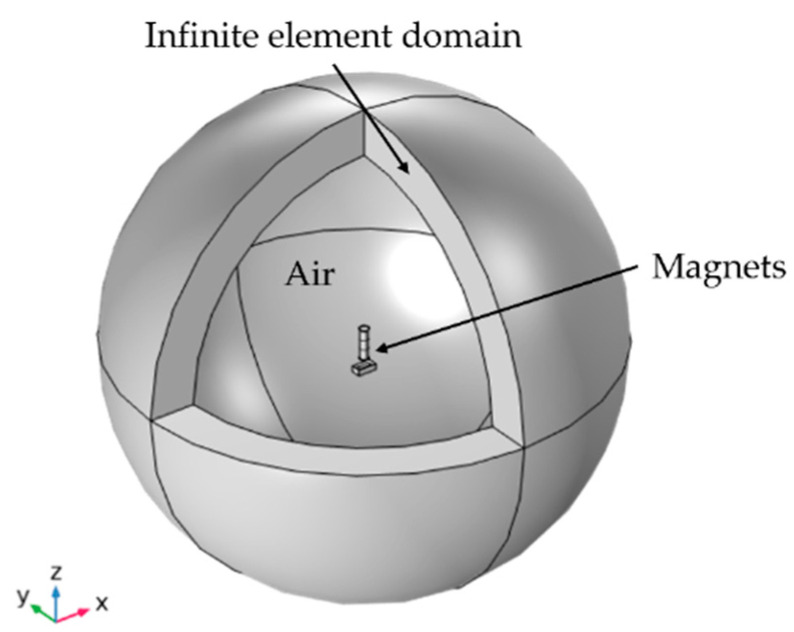
FEM model setup for calculating the magnetic forces between tip and external magnet.

The magnetic force components acting vertically (F_x_) and transversely (F_y_) on the tip magnet were simulated using the AC/DC module of COMSOL Multiphysics. The computational model consists of tip and external magnet volumes surrounded by air. An infinite element domain node is defined to the outside of the spherical volume (Figure 13), through which COMSOL approximates an infinitely large domain. This reduces the effects of artificial boundaries on the fields in the region of interest [45]. Unstructured triangular and tetrahedral mesh types are utilized for meshing, reaching an average element quality of 0.7. The simulated force values are subsequently used in the boundary force condition to excite the harvester under resonance.

**Figure 14:**
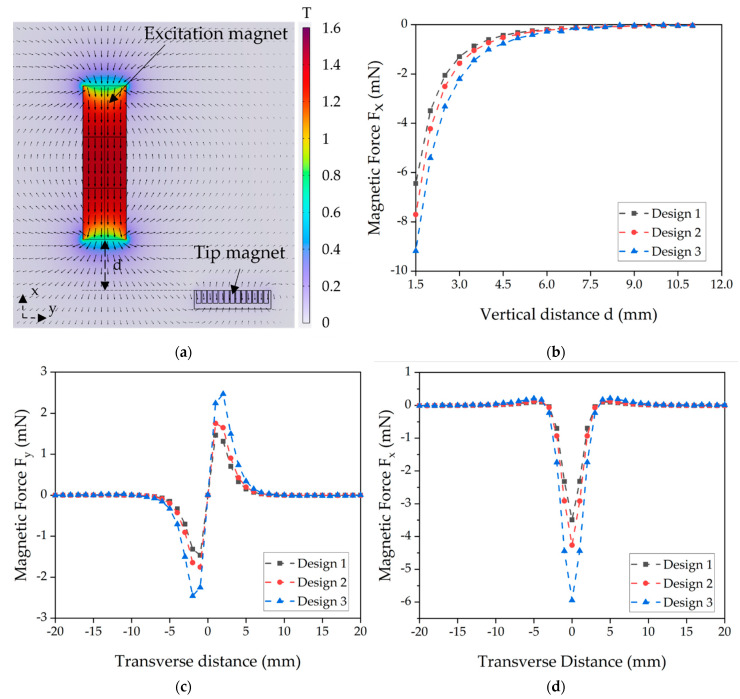
Magnetostatic FEM simulations. (**a**) Magnetic flux density distribution of a design 3 magnet and an excitation magnet. (**b**) Simulated vertical magnetic forces F_x_ for different distances between harvester and excitation magnet. (**c**) Transverse force F_y_ and (**d**) vertical force F_x_ during movement of the tip magnet in vertical direction with constant vertical distance of d = 2 mm.

The magnetic flux density distribution of the micromagnet array of harvester design 3 and the excitation magnet is exemplarily shown in Figure 14a. The direction of magnetization of the harvester magnets is chosen to cause a repulsive force with respect to the excitation magnet. For the harvester located opposite the excitation magnet (zero transverse distance), the values of the vertical magnetic force F_x_ at different vertical distances d for all three harvester designs are plotted in Figure 14b. As anticipated, an increase in magnetic volume results in higher excitation forces.

During the rotational motion of the experimental wheel setup, the magnets pass each other transversely. The respective magnetic forces between the excitation magnet and the tip magnet moving in y-direction are shown in Figure 14c for the transverse component F_y_ and in Figure 14d for the vertical magnetic force F_x_, considering a constant vertical distance d = 2 mm.

**Figure 15:**
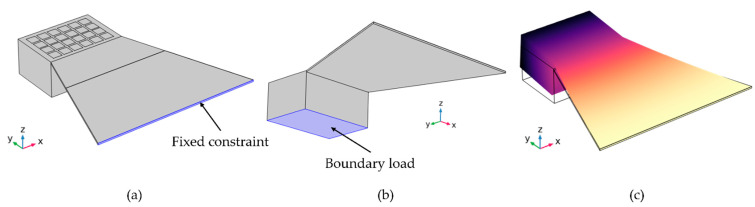
(**a**) Fixed constraint and (**b**) boundary load conditions applied to the geometries. (**c**) First deformation mode of the design 1 harvester.

To verify the experimentally observed open-circuit voltage, first, the resonance frequencies of the three harvester designs were simulated by performing eigenfrequency analysis. (The calculated values were already summarized in Table 4). Open-circuit voltage output with respect to vertical distance d was simulated in a frequency domain model. In the latter, the clamped end of the cantilever was fixed (Figure 15a) and the total force boundary load applied to the bottom surface of the tip in positive z-direction, as illustrated in Figure 15b. The poling direction of the piezoelectric domain is in z-direction. An external excitation force of varying magnitude, according to Figure 14b, was exerted at resonance frequency on the tip magnet to simulate different distances d. Subsequently, the model calculates the voltage across the piezoelectric AlN layer resulting from the induced stress σ. (The material properties are given in Section 2 in Table 1 and Table 2). Additionally, for the frequency analysis study, isotropic structural loss factor damping η=2ζ at the resonance frequency [46] was considered. The mechanical damping ζ=1/2Q of the model was calculated by the experimentally determined quality factor Q (using Equation (3), deduced from frequency sweep curves as shown in Figure 11a.

**Figure 16:**
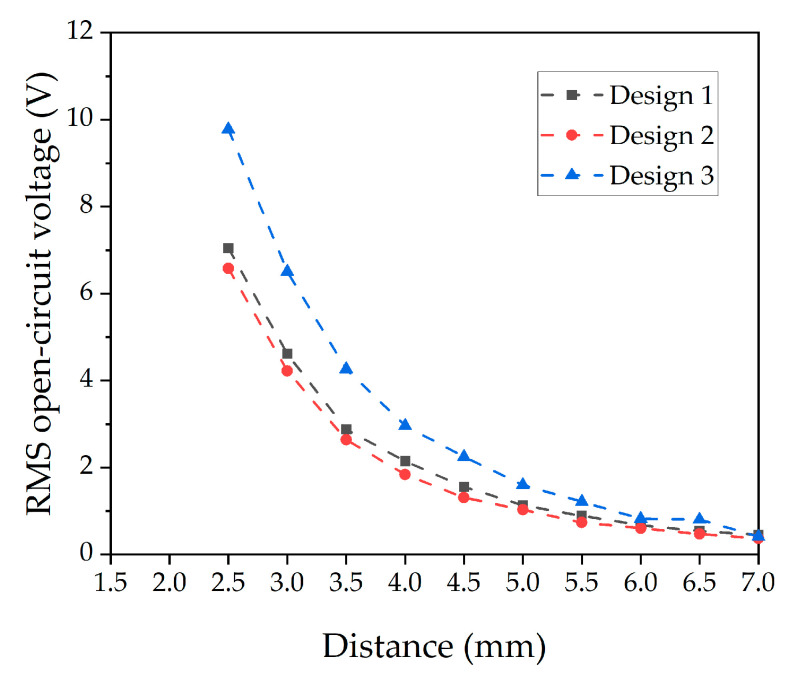
Open-circuit voltage of the three harvester designs simulated at varying the distance d between the magnets.

The relevant bending mode shape used for calculating the resonance frequencies (Table 4) is shown in Figure 15c. The frequency domain simulation was conducted for the same separation distances as used in the experimental measurements. The resulting open-circuit voltages are displayed in Figure 16. Both the general trend of the simulated graphs as well as the maximum voltages are in good agreement with the measured data shown in Figure 12a. Deviations most likely result from simplifications in the static model which, for example, does not consider dynamic processes such as twisting of the cantilever. Due to the reasonable agreement between the predicted and experimental results, the static simulation appears suitable to estimate the performance expected from a specific harvester design and to identify design trends.

**Figure 17:**
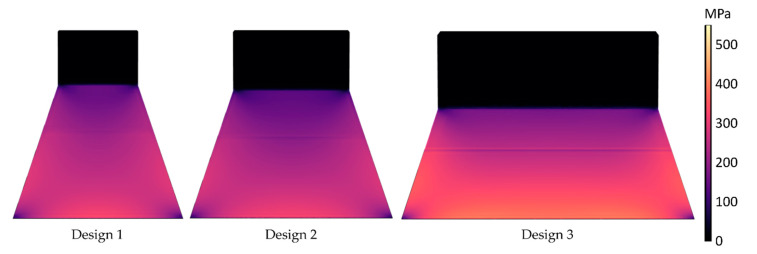
FEM simulated von Mises stress contour plots for the three harvester designs by applying a force (Figure 14d) at distance of 2 mm.

In comparison, design 3 generates higher output voltage than the other two designs. The larger magnetic tip volume of the design results in a higher magnetic force exerted on the tip mass, as shown in Figure 14b. Accordingly, more stress is induced in the piezoelectric material, leading to a higher voltage. However, from FEM simulations (Figure 17), the induced stress is below 550 MPa when a force (Figure 14d) at a distance of 2 mm is applied, which is significantly lower than the fracture strength of the poly-silicon cantilever [47]. This gives a first indication that transverse forces contribute to the failure of some devices at high magnetic excitation.

### 5.2. Mechanical Excitation in Resonance

**Figure 18:**
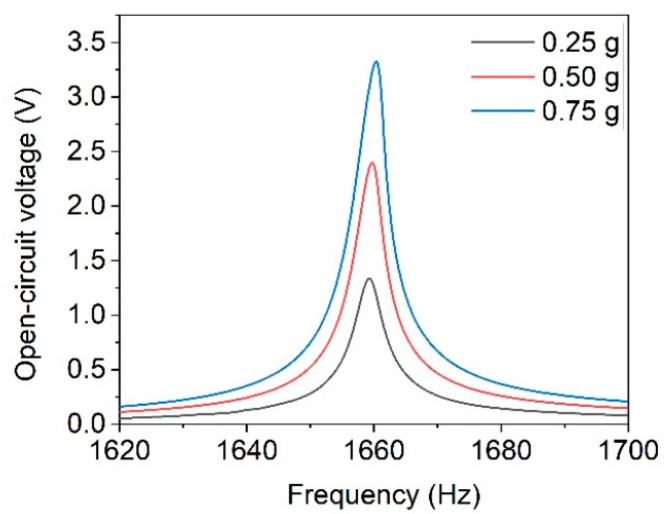
Open-circuit voltage in dependence of excitation frequency of design 3 harvester at varying excitation accelerations.

A purely mechanical excitation without external magnets might be desired, depending on the application requirements. Thus, the performance of the harvesters under mechanical excitation in resonance is evaluated as follows. Measurements were performed under open-circuit conditions and an optimal load applied. Since the acceleration values of common ambient sources of vibration are well below 1 g [16], the tests were executed with acceleration values of 0.25 g, 0.50 g and 0.75 g. The resulting resonance curves for design 3 are depicted in Figure 18. The slight shift of the resonance frequency and the increasingly asymmetric resonance curve are indications for hardening. Hardening is commonly observed for cantilever-based harvesters with AlN and is associated to non-linearity responses, which become more prominent with increasing excitation [48,49].

**Figure 19:**
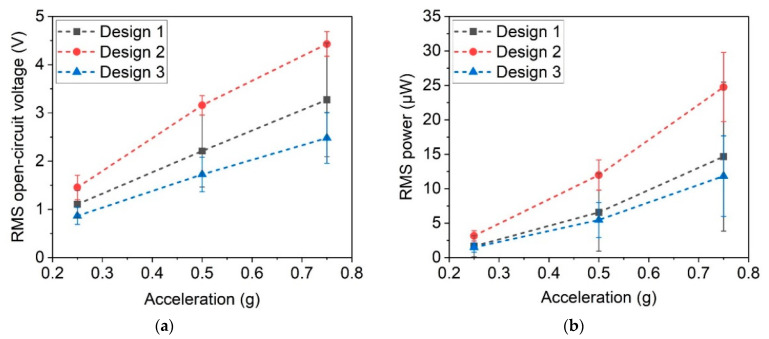
(**a**) Open-circuit voltage and (**b**) power output at optimal resistance for the different harvester designs in resonance for accelerations of 0.25 g, 0.5 g and 0.75 g.

From the resonance curves, as shown in Figure 18, the open-circuit voltage was determined, and the power output was calculated in resonance for all three harvester designs. As expected, the open-circuit voltage, in Figure 19a, and the power output, in Figure 19b, increase with higher excitation acceleration. An open-circuit voltage up to 4.43 V RMS and power of 24.75 µW RMS were achieved. Mechanical excitation design 2 exhibits better performance than design 3, contrary to the case of magnetic excitation (see Section 5.1). The large tip magnet of design 3 leads to high forces of several mN in the case of magnetic excitation, while the cantilever is mechanically stiff due to its wide base. Accordingly, a mechanical excitation, which is independent of the magnetic volume, leads to a higher output for the softer design 2.

### 5.3. Frequency Up-Conversion

It is well known that vibrational energy harvesters exhibit best performance when operated under resonant conditions. Resonance frequencies of MEMS energy harvesters are typically in the range of hundreds to thousands of Hz. However, in many applications, low-frequency sources < 100 Hz from ambient vibrations, shocks or rotating machinery parts, e.g., tires, need to be harvested [50,51]. A common scheme to access low frequencies well below resonance is frequency up-conversion [25,52]. In this work, frequency-up conversion is realized both through excitation by magnetic plucking from a rotational motion and through excitation by mechanical shocks. The output under mechanical and magnetic frequency up-conversion is characterized and compared to one another. For this study, design 2 harvesters were used since this design exhibited best performance in the investigations above, considering both generated output and mechanical stability.

For power measurements, the optimal load resistance as determined for the resonant case was used. Since harvesting takes place in self-resonance between the plucking events, it is reasonable to assume that the frequency dependent components of the internal resistance are similar to those in the resonant case.

Mechanical excitation was provided by the shaker setup (Figure 9b) facilitating square-pulse signals with varying frequency and constant acceleration of 51 g. The duty cycle of the pulses was kept constant at 10%.

**Figure 20:**
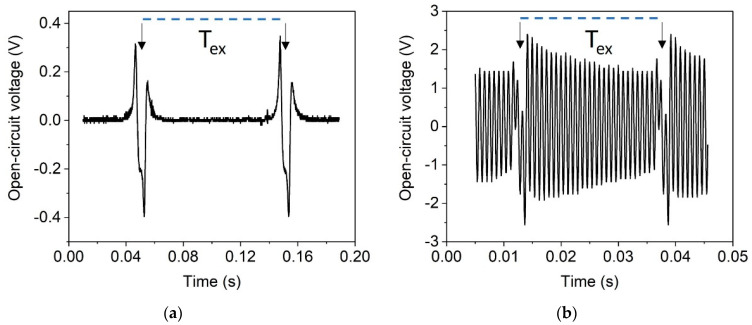
Time-dependent open-circuit voltage signal for a harvester of design 2 excited by a single magnet in rotary motion (see Figure 8b). Single excitation events are highlighted with arrows. The period length T_ex_ of the external excitement magnet is highlighted with a dotted line. (**a**) Harvester response at low excitation magnet velocity (v_r_ = 1.55 m/s); (**b**) magnetic plucking at v_r_ = 6.27 m/s, corresponding to an excitation frequency of 39.9 Hz.

For magnetic excitation, the single-magnet pole wheel (Figure 8d) was used. In this rotational setup, the excitation frequency determines the tangential velocity $v_{r}=2\pi \mathrm{fr}$ of the excitation magnets, where f is the rotation frequency and r the distance between magnet and rotation axis. At low velocities, the stray field of the passing excitation magnet dampens the ring-down of the cantilever. Thus, the latter performs no free oscillation after the plucking event, as depicted in Figure 20a. The observed voltage response indicates that the tip magnet is not decoupled from the field of the excitation magnet after initial deflection. The resulting slow release of the harvester s suppresses its free oscillation. At sufficiently high velocities, magnetic plucking is observed as the stray field of the excitation magnets vanishes fast enough after deflection to enable free oscillation between the excitation events. [22,25,53]. As depicted in Figure 20b, the harvester oscillates in this case at its resonance frequency $f_{0}$ = 1210 Hz, because of a low-frequency excitation with $f_{\mathrm{ex}}$ = 39.9 Hz, representing effective frequency up-conversion.

**Figure 21:**
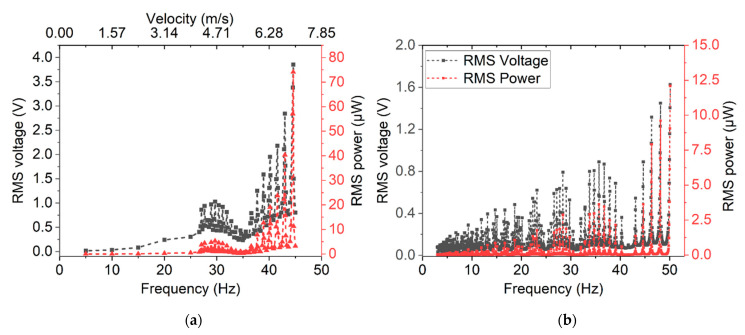
RMS voltage and calculated RMS power output achieved for design 2 harvesters at different excitation rates: (**a**) magnetic excitation with rotating pole wheel; (**b**) mechanical excitation at a constant acceleration of 51 g.

The measured RMS voltage and power for magnetic and mechanical excitation with respect to excitation frequency is shown in Figure 21a and Figure 21b, respectively. Within the investigated frequency range, a 74 µW RMS power output was achieved for magnetic excitation and 12.2 µW for mechanical excitation. The difference is discussed in Section 6.

As reasoned above, in the case of magnetic excitation, frequency up-conversion does not occur for excitation magnet velocities below approximately $v_{r}$ = 3 m/s. This lower frequency limit is not observed in the case of mechanical excitation, since the free oscillation is not dampened after excitation. Maxima occurring in power and voltage output depending on excitation frequency are well-known for frequency up-conversion, as also discussed in [22]. They occur due to in-phase excitation when the resonance frequency of the cantilever equals an integer multiple of the excitation frequency

(5) $$f_{0}={n\;f}_{\mathrm{ex}}$$

where $f_{\mathrm{ex}}$, n and $f_{0}$ represent the excitation frequency, an integer, and the resonance frequency of the cantilever, respectively.

### 5.4. Energy Storage Demonstrator

Output power achieved under optimal load and in resonance do not necessarily represent the performance of a vibrational energy harvester under real world conditions. Thus, rectification and storage of the AC output of a design 2 harvester excited by magnetic plucking was studied using a custom-designed ASIC. The harvester, ASIC and capacitors were integrated on a single PCB module (already depicted in Figure 10).

**Figure 22:**
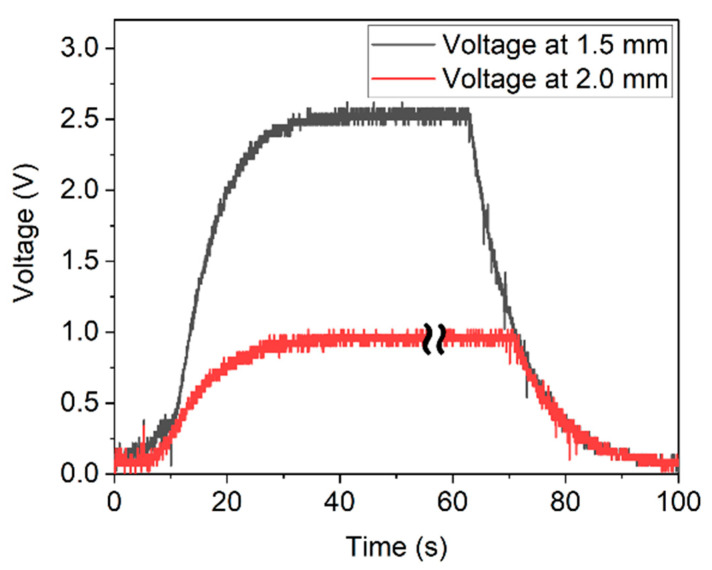
Charging and discharging curve of the capacitors resulting from magnetic plucking at distances of d = 2.0 mm (red curve) and d = 1.5 mm (black curve). Some data are skipped in the plateau of the red curve to align the timeline of both graphs.

Excitation of the harvester was conducted at a rotation speed of 6.81 m/s, corresponding to a frequency of 43.33 Hz. The measurements were performed at distances of d = 2.0 mm and d = 1.5 mm between excitation magnet and harvester (Figure 22). The generated power was sufficient to drive the ASIC and to fully charge the capacitors within 30 s. A maximum voltage of 0.9 V and 2.5 V for 2.0 mm and 1.5 mm were obtained, respectively (see Figure 22). Accordingly, up to ≈8.75 µJ were stored in the capacitors.

## 6. Discussion

Both in frequency up-conversion and in resonance magnetic excitation yielded significantly higher maximum output of the harvesters. This can be explained by the fact that magnetic interaction between two NdFeB micromagnets can generate non-contact forces in the range of several mN over distances of a few millimeters. To achieve comparable forces with mechanical excitation, high acceleration is required. For example, a force of 9 mN exerted on the tip mass of a harvester of design 2 is equivalent to an acceleration of approximately 200 g. Such acceleration occurs in car wheels at a velocity of 90 km/h [51]. It is about two orders of magnitude above the range of acceleration values commonly available from environmental sources, such as infrastructure constructions, home appliances or commercial devices [15,16,41]. Thus, magnetic force coupling allows for high excitation forces at low-g movements or rotations. Accordingly, using magnetically excited frequency up-conversion, considerable amounts of energy can be harvested at moderate excitation conditions. Such a magnet-based excitation scheme is enabled by the novel PowderMEMS fabrication technique since the wafer-level integrated rare earth magnets presented in this study are a unique feature for MEMS. Up to now, either non-microscale systems were used for magnetic harvesting or hybrid integration was required to equip a MEMS device with a rare-earth magnet [22,29,31,54].

Besides wafer-level integration of magnets, PowderMEMS allows for increasing the tip mass at constant volume using powders of dense materials, such as NdFeB (7.6 g/cm^3^), tantalum (16.7 g/cm^3^) or tungsten (19.2 g/cm^3^). Considering a powder-based structure with a filling factor between 50–70%, such materials achieve significantly higher densities than bulk silicon (2.3 g/cm^3^). To compare the performance under mechanical excitation of the devices presented in this study with other works, the output power reported here is converted to power densities. To calculate area power densities, significant for MEMS fabrication costs, the area of the cantilever and tip mass is considered, as in other reports. Furthermore, for better comparison of different vibrational harvesters, those area densities were normalized to the applied mechanical acceleration level. Table 5 summarizes different piezoelectric vibrational harvesters which were excited mechanically in resonance. The device presented in this study exhibits power densities which are among the highest reported up to now. This result can be mainly attributed to two reasons. First, an AlN layer of 2 µm thickness is used, exceeding the active layer thickness of comparable devices [55] (for example, PZT as the piezoelectric layer for energy harvesting suffers from its high permittivity, usually achieving a lower figure of merit than AlN [48,55]). Secondly, excepting [37], the devices in Table 5 feature silicon-based tip masses. Due to the low density of Si, the inertial mass limits the achievable power densities.

Regarding the long-term stability of the devices presented here, a detailed investigation needs to be conducted in future work. However, the individual components are known to be highly reliable, such as AlN thin films [56] and polysilicon cantilevers [57]. Regarding the integrated micromagnets, it was demonstrated that during storage under ambient conditions for two years, no decrease in performance is observed [58]. A similar device (featuring AlScN instead of AlN) exhibited no degradation when excited in resonance over the course of 150 h [31].

## 7. Conclusions and Outlook

A fully wafer-level fabricated piezoelectric MEMS energy harvester with integrated rare earth permanent magnetic structures was presented. Under magnetic excitation, the device achieves a power output of up to 140 µW in resonance and up to 74 µW for frequencies < 50 Hz in frequency up-conversion. For mechanical acceleration excitation, 24.75 µW were achieved at 0.75 g in resonance and an output of 12.2 µW was generated in frequency up-conversion at 51 g and 50 Hz. The output at mechanical excitation is among the highest reported for comparable MEMS energy harvesters, although magnetic excitation delivers even higher outputs in the chosen excitation setups. This is since magnetic tip masses enable high excitation forces from low-g and/or low-frequency mechanical sources by magnetic force coupling. However, for a purely mechanical excitation, the PowderMEMS technique offers the advantage of high tip mass densities compared to state-of-the-art bulk silicon. Thus, the wafer-level integration of rare earth micromagnets and dense materials allows the design of novel MEMS energy harvesters with low resonance frequencies, a high power output and contactless magnetic force coupling.

Future work aims for the replacement of AlN with AlScN [67], which is expected to increase the output by at least a factor of three [31,68]. The utilization of wafer-level vacuum packaging will further increase the q-factor and potentially the electrical output [69]. Additionally, the integrated magnets enable the active tuning of the resonance frequency using dedicated magnetic configurations [28,52]. The device designs will be optimized by more sophisticated FEM simulations in combination with the novel PowderMEMS technique in terms of output and mechanical stability for specific application scenarios.

## Figures and Tables

**Table 1:** AlN piezoelectric material properties used for FEM simulations.

Elasticity matrix (c^E^), ×10^11^ Pa	$\left[\begin{array}{cccccc} 4.1 & 1.49 & 0.99 & 0 & 0 & 0\\ 1.49 & 4.1 & 0.99 & 0 & 0 & 0\\ 0.99 & 0.99 & 3.89 & 0 & 0 & 0\\ 0 & 0 & 0 & 1.25 & 0 & 0\\ 0 & 0 & 0 & 0 & 1.25 & 0\\ 0 & 0 & 0 & 0 & 0 & 1.305 \end{array}\right]$
Coupling matrix (e), C/m^2^	$\left[\begin{array}{cccccc} 0 & 0 & 0 & 0 & -0.48 & 0\\ 0 & 0 & 0 & -0.48 & 0 & 0\\ -0.58 & -0.58 & 1.55 & 0 & 0 & 0 \end{array}\right]$
Relative permittivity (ε^s^)	$\left[\begin{array}{ccc} 9 & 0 & 0\\ 0 & 9 & 0\\ 0 & 0 & 9 \end{array}\right]$
Density (ρ), kg/m^3^	3300

**Table 2:** Mechanical material properties used for FEM simulations.

Material	Relative Permittivity	Density (kg/m^3^)	Young’s Modulus (GPa)	Poisson’s Ratio
Poly-Silicon	4.5	2320	160	0.22
Silicon oxide	4.2	2200	70	0.17
Silicon	11.7	2329	170	0.28

**Table 3:** Summary of the geometric parameters of the harvester designs.

	Design 1	Design 2	Design 3
**Tip mass**	a_1_ = 1.5 mm	b_1_ = 1.9 mm	c_1_ = 3 mm
	a_2_ = 1 mm	b_2_ = 1 mm	c_2_ = 1 mm
**Micromagnet** **(single array element)**	a = 0.18 mm	b = 0.18 mm	c = 0.18 mm
**Cantilever**	a_3_ = 0.85 mm	b_3_ = 0.8 mm	c_3_ = 0.55 mm
	a_4_ = 2.45 mm	b_4_ = 2.25 mm	c_4_ = 1.5 mm
	a_5_ = 3.2 mm	b_5_ = 3.5 mm	c_5_ = 4 mm
**Cantilever volume including tip**	0.924 mm^3^	1.175 mm^3^	1.652 mm^3^
**Electrode area**	4.22 mm^2^	4.38 mm^2^	3.49 mm^2^

**Table 4:** Resonance frequencies, Q-factors, calculated damping ratios and internal resistances for the different designs in the case of magnetic excitation.

Sample	$f_{0}\;(\mathrm{Measured})\;(\mathrm{Hz})$	$f_{0}\;(\mathrm{Simulated})\;(\mathrm{Hz})$	Q-Factors	Damping Ratios	Internal Resistance (kΩ)
Design 1	1194.84 ± 9.55	1184	132–201	0.00379–0.0249	166 ± 7
Design 2	1230.58 ± 15.54	1218	339–391	0.00147–0.00128	186 ± 13
Design 3	1674.49 ± 4.00	1684.5	330–335	0.00152–0.00149	180 ± 20

**Table 5:** Comparison of power output and power densities for mechanically excited energy harvesters.

	Piezoelectric Material	Resonance Frequency (Hz)	Excitation Acceleration (g)	Maximum Power Output (µW)	Areal Power Density (µW/mm^2^)	Normalized Areal Power Density (µW/mm^2^g^2^)
Fang et al. [59]	PZT	608	1	2.16	1.8	1.8
Elfrink et al. [55]	AlN	572	2	60	1.99	0.49
Lei et al. [60]	PZT	235	1	14	0.39	0.39
Shen et al. [61]	PZT	461.15	2	2.15	0.84	0.21
Dow et al. [62]	AlN	572	2	34.78	0.95	0.24
Park et al. [63]	PZT	528	0.39	1.1	0.6	4.02
Aktakka et al. [37]	PZT	154	1.5	205	4.18	1.86
Xu et al. [64]	PZT	-	1	37.1	1.04	1.04
Andosca et al. [65]	AlN	58	0.5	32	0.489	1.96
Muralt et al. [66]	PZT	870	2	1.4	1.45	0.36
This work (design 2)	AlN	1230	0.75	24.75	3.1	5.5
